# Supplementary material for: Accumulated local effects and graph neural networks for link prediction
Source: Sci Rep. 2026 Feb 12;16:8574. doi: 10.1038/s41598-026-39000-w (PMC12976273; doi:10.1038/s41598-026-39000-w)
Supplement: Supplementary file 1 — Supplementary Information. [file 41598_2026_39000_MOESM1_ESM.pdf]

Supplementary Material for Accumulated Local Effects and Graph Neural Networks for link prediction

Paulina Kaczyńska[1,2*]**, Julian Sienkiewicz**[3,4]**, and Dominik Ślęzak**[1]

[1]University of Warsaw, Faculty of Mathematics, Informatics and Mechanics, Institute of Informatics, Banacha 2, 02-097 Warsaw, Poland
[2]Polish Academy of Sciences, Institute of Fundamental Technological Research, Pawińskiego 5B, 02-106, Warsaw, Poland
[3]Warsaw University of Technology, Faculty of Physics, Koszykowa 75, 00-662 Warsaw, Poland
[4]Warsaw University of Technology, Centre for Credible AI, Rektorska 4, 00-614 Warsaw, Poland
[*]pm.kaczynska@student.uw.edu.pl

ABSTRACT

In this supplementary material to the article "*Accumulated Local Effects and Graph Neural Networks for link prediction*", we show the Average Root Mean Square Error (RMSE) between ALE and the underlying relation in the synthetic dataset for different values of graph sparsity as well as examine the difference between RMSE and exact predictions for specific combinations of the number of modified nodes and the number of sampled edges.

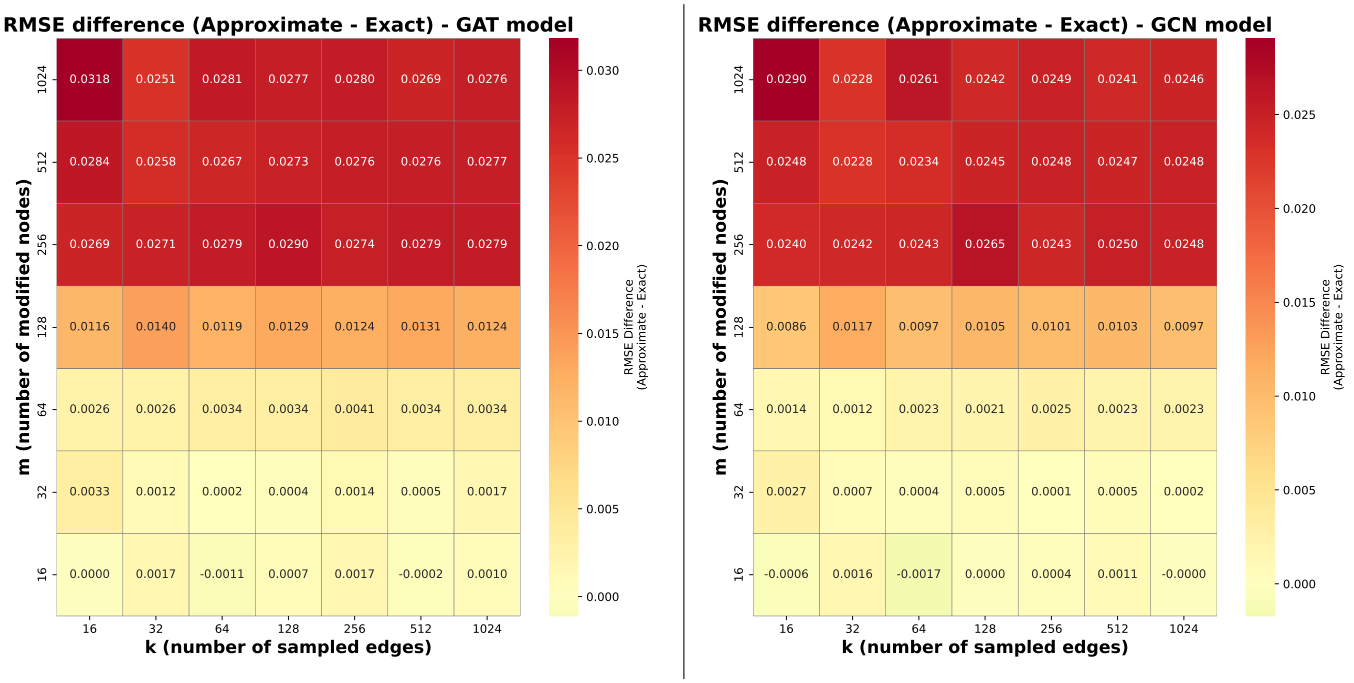

Figure S1. Heatmaps of Root Mean Square Error difference between the approximate and exact method for GAT model (left) and GCN model (right) versus the number of sampled edges k (horizontal axis) and the number of modified nodes m (vertical axis).

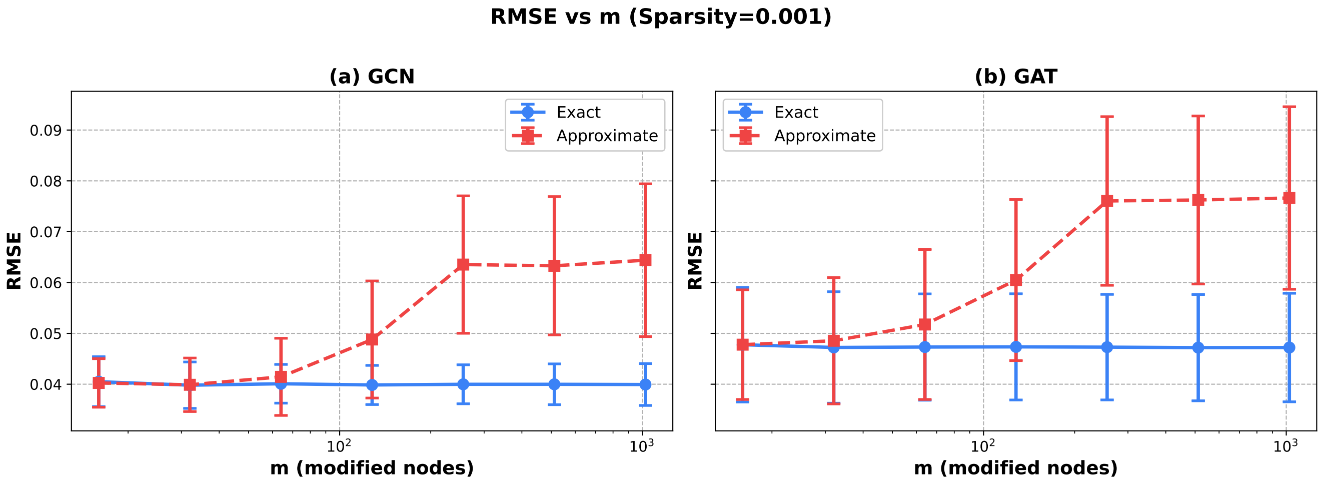

Figure S2. Average Root Mean Square Error between ALE and the underlying relation in the synthetic dataset versus the number of modified nodes m for graph sparsity equal to 0.001 and for (a) GCN model, (b) GAT model. The errorbars correspond to the standard deviation across explanations for different models.

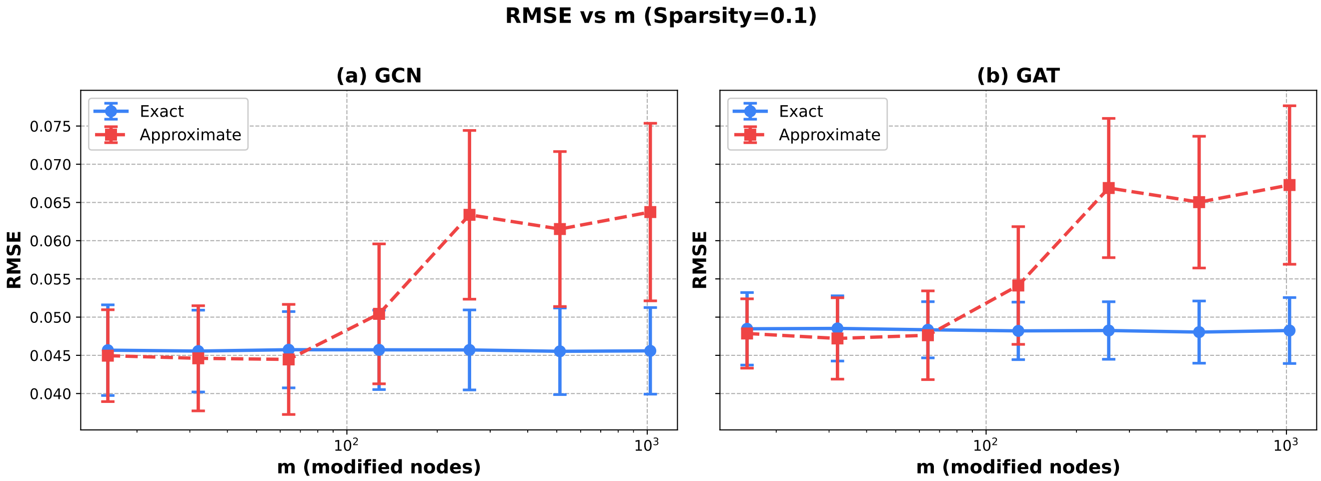

Figure S3. Average Root Mean Square Error between ALE and the underlying relation in the synthetic dataset versus the number of modified nodes m for graph sparsity equal to 0.1 and for (a) GCN model, (b) GAT model. The errorbars correspond to the standard deviation across explanations for different models.
